# p53-driven lipidome influences non-cell-autonomous lysophospholipids in pancreatic cancer

**DOI:** 10.1186/s13062-022-00319-9

**Published:** 2022-03-08

**Authors:** Alessio Butera, Micaela Roy, Carlotta Zampieri, Eleonora Mammarella, Emanuele Panatta, Gerry Melino, Angelo D’Alessandro, Ivano Amelio

**Affiliations:** 1 Department of Experimental Medicine, TOR, University of Rome Tor Vergata, 00133 Rome, Italy; 2 University of Colorado Anschutz Medical Campus, Aurora, CO 80045 USA; 3 School of Life Sciences, University of Nottingham, Nottingham, UK

**Keywords:** p53, Pancreatic cancer, Lipid metabolism, Phospholipase

## Abstract

Adaptation of the lipid metabolism participates  in cancer pathogenesis, facilitating energy storage and influencing cell fate and control of molecular signalling. The tumour suppressor protein p53 is a molecular hub of cell metabolism, supporting antioxidant capabilities and counteracting oncogene-induced metabolic switch. Despite extensive work has described the p53-dependent metabolic pathways, a global profiling of p53 lipidome is still missing. By high-throughput untargeted lipidomic analysis of pancreatic ductal adenocarcinoma (PDAC) cells, we profile the p53-dependent lipidome, revealing intracellular and secreted lysophospholipids as one of the most affected class. Lysophospholipids are hydrolysed forms of phospholipids that results from phospholipase activity, which can function as signalling molecules, exerting non-cell-autonomous effects and instructing cancer microenvironment and immunity. Here, we reveal that p53 depletion reduces abundance of intracellular lysophosphatidyl-choline, -ethanolamine and -serine and their secretion in the extracellular environment. By integrating this with genomic and transcriptomic studies from in vitro models and human PDAC patients, we identified potential clinically relevant candidate p53-dependent phospholipases. In particular *PLD3*, *PLCB4* and *PLCD4* expression is regulated by p53 and chromatin immunoprecipitation followed by deep sequencing (ChIP-seq) indicates a direct transcriptional control on their chromatin accessible genomic loci. Consistently, *PLD3*, *PLCB4* and *PLCD4* expression correlates with p53 mutational status in PDAC patients, and these genes display prognostic significance. Overall, our data provide insights into lipidome rewiring driven by p53 loss and identify alterations of lysophospholipids as a potential molecular mechanism for p53-mediated non-cell-autonomous molecular signalling that instructs cancer microenvironment and immunity during PDAC pathogenesis.

**Supplementary Information:**

The online version contains supplementary material available at 10.1186/s13062-022-00319-9.

## Introduction

Pancreatic cancer remains one of the deadliest malignancies worldwide, with an extremely low 5-year survival rate (< 5%) [1]. Among the diverse types of pancreatic cancer, pancreatic ductal adenocarcinoma (PDAC) is the most represented. At a genomic level, initial activating mutation in KRAS is the key step at the basis of tumour initiation, driving the formation of a histologically differentiated pancreatic intraductal neoplasia (PanIN) [2]. The subsequent inactivating mutations of TP53, occurring in > 70% of cases, usually drives the final steps of malignancy towards the formation of a metastatic tumour [1, 2]. Throughout the tumorigenic process, PDAC cells undergo an extensive rewiring of metabolic pathways, a well-established hallmark of cancer, which is indispensable to help to sustain their own growth and to adapt to microenvironmental stress conditions [3, 4]. Generally, metabolic reprogramming proceeds with the acquisition of driver mutations. For example, mutant *KRAS* and *MYC* are known to trigger, beyond glucose and amino acid, also the rewiring of lipid pathways in a variety of tumour models [5–10].

In addition to the crucial role in maintenance of genomic integrity [11, 12], the tumour suppressor p53 is known to regulate many pathways of intracellular metabolism [13–16]. p53 dictates a tumour suppressive programme by controlling mitotic and oncogenic signals that converge on the beta oxidation of free fatty acids, glucose and amino acids metabolism [17, 18]. Mutant forms of p53 have also been associated to gain of function effects [19–22], whose roles have been ascribed to regulation of tumour microenvironment and cellular metabolism [23, 24], including mevalonate pathway [25]. In the context of lipid metabolism, recent evidence from genetically engineered mouse models indicates that mevalonate pathway is selectively activated not only in p53 mutant tumours, but also p53^null^ tumours. The tumour suppression activity of p53 is partially exerted by negatively and indirectly regulating the aforesaid pathway, by influencing the post-translational processing of SREBP-2 [26].

While the role of wild-type p53 in the regulation of specific metabolic pathways of cancer cells including cholesterol and fatty acid metabolism has been dissected, a global and effective map of the changes in the lipidome profile in the context of PDAC is still missing. Here, by using a cell line derived from a mouse model of pancreatic adenocarcinoma with the pancreas-specific expression of *KRAS* (LSL-*KRAS*^G12D^) and an inducible short hairpin (sh) RNA targeting the endogenous *Trp53*, we attempt to provide a fine and novel map of lipid changes occurring after the loss of p53, in the process of pathogenesis of PDAC. By integrating the lipidomic profiling with the transcriptomic of in vitro models and PDAC patients we identify a mechanistic link in the p53 regulation of lysophospholipids. Our findings suggest an important involvement of p53 in remodelling the lipidome of pancreatic cancer cells and may direct future studies in this field.

## Results

### p53 remodels the lipidome of pancreatic cancer cells

**Fig. 1:**
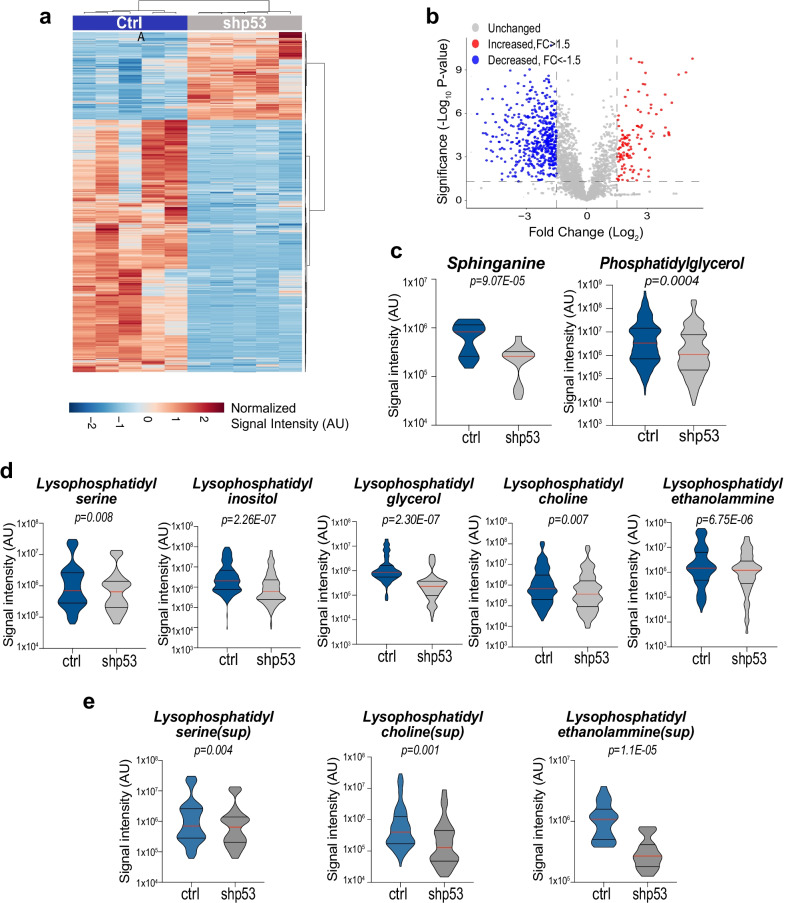
p53 rewires pancreatic cancer lipidome. **a**, **b** Heatmap and Volcano plot showing global changes of lipids upon depletion of p53. For Volcano plot a *p* value < 0.05 and a − 1.5 > fold change > 1.5 were used as threshold. Lipid abundancies are sown as signal intensities (AU: arbitrary units). N = 5 biological replicates per condition. **c**, **d** Violin plots showing the significantly modulated lipid classes after p53 knockdown. *p* values are indicated. **e** Violin plots showing the significantly modulated lipid classes in the conditioned medium after p53 knockdown. *p* values are indicated

To explore the role of p53 in remodelling the lipid profile of pancreatic cancer, we carried out a global untargeted lipidomic profiling in a cell line (KPshp53) derived from mouse PDAC, with the pancreas-specific expression of *KRAS* (LSL-*KRAS*^G12D^) and a doxycycline-regulated short hairpin (sh)RNA targeting wild-type p53 expression. Lipidomic results indicate that loss of p53 extensively remodels the lipidome of pancreatic cancer cells (shp53, *p* < *0.05*) (Fig. 1a, b). By clustering lipid species in biological classes, we observed that sphinganine, phosphatidylglycerol (PG), lysophosphatidylserine (LPS), lysophosphatidylcholine (LPC), lysophosphatidylethanolammine (LPE), lysophosphatidylinositol (LPI) and lysophosphatidylglycerol (LPG) were the most significantly altered classes, displaying a dramatic reduction of their abundancies upon p53 depletion (Fig. 1c, d and Additional file 1: Fig. S1). Conversely, other lipid classes including glucosylceramides, sphingosines, diacylglycerols, triglycerides, ceramides, hexosyl-ceramides, palmitate, and phospholipids were generally not comprehensively affected by p53 loss, despite displaying decreases of specific species (Additional file 1: Fig. S2a). Lysophospholipids are a class of lipids exerting signalling roles in a cell-autonomous and non-cell-autonomous manner [27], and can function also as signalling molecules in the microenvironment. Hence, we conducted a parallel global untargeted lipidomic profiling of conditioned media from KPshp53 cells. Consistently, we observed a massive reduction of lysophospholipid species (LPS, LPC, LPE) in the extracellular environment of doxycycline treated KPshp53 cells (p53 silenced) (Fig. 1e and Additional file 1: Fig. S2b). Hence, overall, these data clearly indicate that p53 has an essential role in controlling the lipidome of pancreatic cancer cells and, in particular, it can exert an important regulation of intracellular and extracellular signalling lysophospholipids.

### p53 regulates production and secretion of lysophospholipidome

**Fig. 2:**
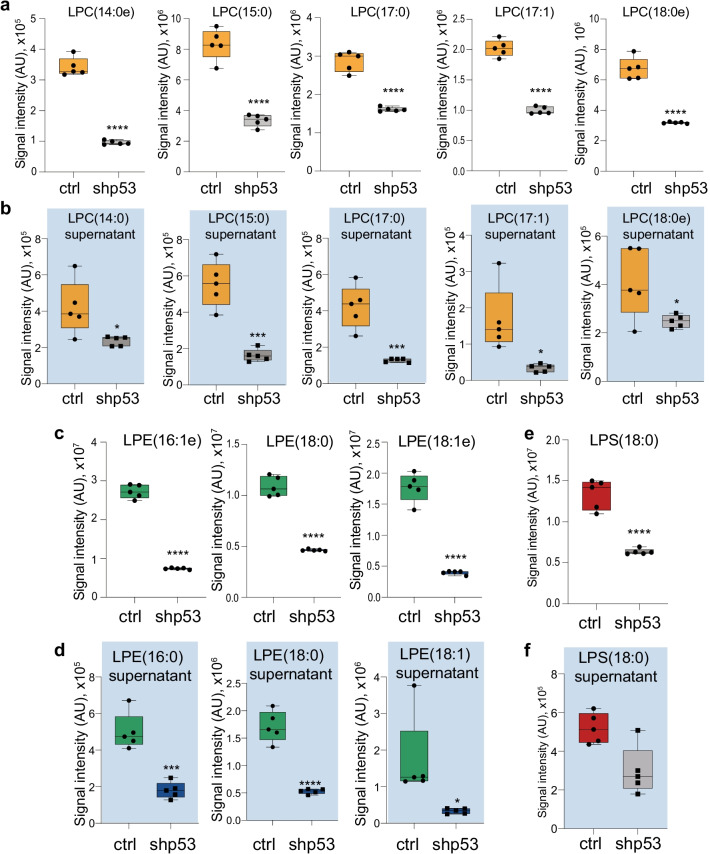
Lysophospholipids are strongly affected by p53 loss. **a**–**f** Box plots showing the most significantly affected lipid species per lysophospholipid class intracellularly (white background, **a**, **c**, **e**) and in the conditioned media (light blue background, **b**, **d**, **f**. Lipid abundancies are shown as signal intensities (AU: arbitrary units). N = 5 biological replicates per condition, *p < 0.05; ***p < 0.001; ****p < 0.0001. LPC, Lysophosphatidylcholine; LPE, Lysophosphatidylethanolammine; LPS, Lysophosphatidylserine

To better define the changes mediated by p53 on the lysophospholipidome, we next conducted a detailed analysis comparing the species displaying a differential abundance following p53 deficiency in the intracellular compartment and in the conditioned media. The most significantly altered LPC, showing consistent reductions, were the 14:0, 15:0, 17:0, 17:1 and 18:0e species (Fig. 2a,b). Our analysis however revealed a larger general cohort of intracellular and extracellular LPC species (Additional file 1: Figs. S1 and S3a), indicating overall the LPC among the mostly affected lysophospholipids. LPC is in general the most abundant class of lysophospholipids in plasma and body fluids and despite a clear dissection of the role of these molecules has not been conducted they appear to participate in cytotoxicity, haemostatis and inflammation [27]. Nanomolar concentrations of LPC can exert chemotactic roles for monocytes and macrophages, while saturated and monosaturated LPC can facilitate production of inflammatory redox oxygen species [28, 29]. Thus, p53 loss dependent reduction of LPC might underlie an immune evasion effect in cancer.

Similar pattern was observed in LPE and LPS classes, where saturated and monosaturated LPE 16:1e, 18:0, 18:1e and LPS18:0 were strongly reduced in the intracellular compartment and in the conditioned media of p53 depleted cells (Fig. 2c–f and Additional file 1: Fig. S4a, b). LPE is the second most abundant lysophospholipids in plasma. LPE can induce an increase of intracellular Ca^2+^ concentration producing proliferative and motilities activities in breast and ovarian cancer cell lines [30]. Remarkably, LPE was also shown to stimulate chemotactic migration and cellular invasion in ovarian cancer cells, indicating non-cell-autonomous properties [31]. LPS is within the less abundant lysophospholipids in the plasma, but it can display potent immunomodulatory activities. G-coupled receptors belonging to the P2Y purineceptor clusters have been identified as LPS receptors and their activation was associated to suppression of T cell and mast cell degranulation [32, 33]. Finally, specific reduction in the intracellular content of LPI and LPG was also detected, indicating a general alterations of lysophospholipids production (Additional file 1: Figure S4c, d).

Overall, these data indicate the p53-dependent lipidome significantly impinge on production and secretion of lysophospholipids. This might represent an unexpected, novel level of regulation exerted by p53 on the tumour microenvironment and immunity.

### p53 transcriptionally regulates phospholipases

**Fig. 3:**
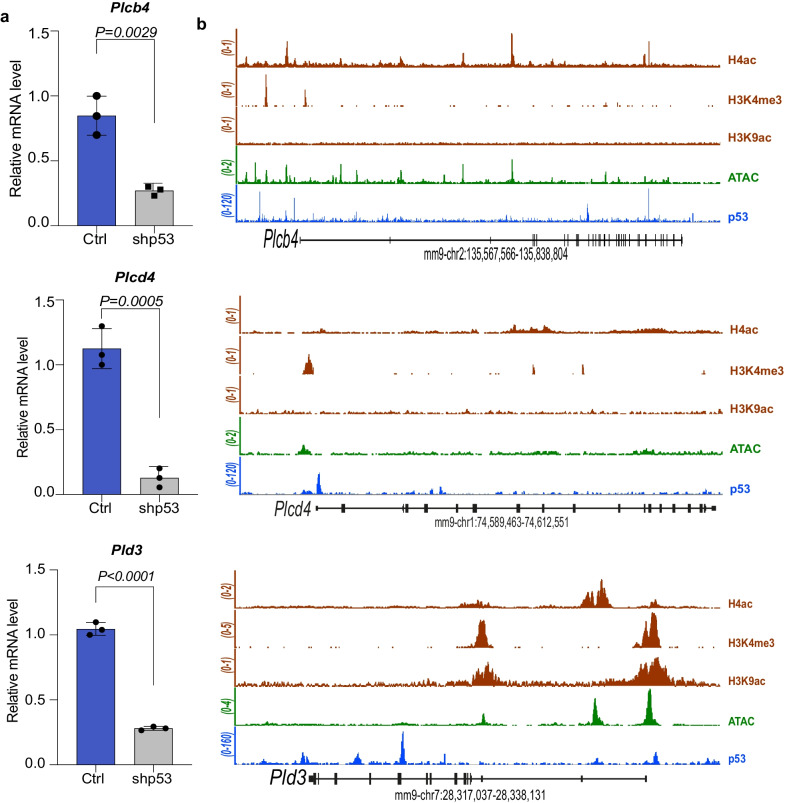
Phospholipases are regulated by p53 in pancreatic cancer cells. **a** Quantitative PCR analysis showing the decreased expression of PLCB4, PLCD4 and PLD3 after p53 silencing in KPshRNA cells. N = 3, *p* values are indicated. **b** ChIP-seq tracks for p53, H4ac, H3K4me3 and K9ac showing gene regions of PLCB4, PLCD4 and PLD3 (GSE63666). PLCB4: Phospholipase C Beta 4, PLCD4: Phospholipase C Delta 4, PLD3: Phospholipase D3

In the last decade cancer genomic sequencing studies have experienced a massive growth, resulting into the formation of datasets containing huge amount of open-access data [34]. By performing a deep analysis of publicly available datasets of pancreatic cancer, we aimed to identify putative phospholipases (PLs) that might account for the observed p53-dependent lysophospholipidome. Through this approach, we selected three putative phospholipases on the basis of the potential regulation by p53 and their clinical relevance for pancreatic cancer. These were the *phospholipase C delta 4* (*PLCD4*), *phospholipase C beta 4* (*PLCB4*) and *phospholipase D 3* (*PLD3*). Interestingly, the expression of the three phospholipases was strongly affected in p53 deficient cells (Fig. 3a), suggesting the existence of molecular axis downstream of p53 function. We next asked whether PLCD4, PLCB4 and PLD3 might be directly regulated by p53 via a transcriptional control. To address our hypothesis, we looked for binding enrichment of p53 on *PLCD4, PLCB4* and *PLD3* genomic loci, querying available ChIP-seq datasets. Notably, we identified several peaks for p53 in the genomic regions of the three phospholipases, which strongly suggests a direct involvement of p53 in their transcription regulation. Furthermore, we observed that p53 peaks broadly overlapped with regions of enhanced chromatin accessibility (ATAC), which were also enriched for permissive histone modifications such as trimethylation of lysine 4 of histone 3 (H3K4me3), acetylation of histone 4 (H4ac) and acetylation of lysine 9 of histone 3 (H3K9ac) (Fig. 3b). Thus, these data indicate a direct molecular axis p53/PLs, that might underlie a transcriptional reprogramming mediated by p53 for the regulation of enzymes involved in lipid metabolism.

### PLCD4, PLCB4 and PLD3 correlates with p53 status and prognosis of pancreatic cancer patients

**Fig. 4:**
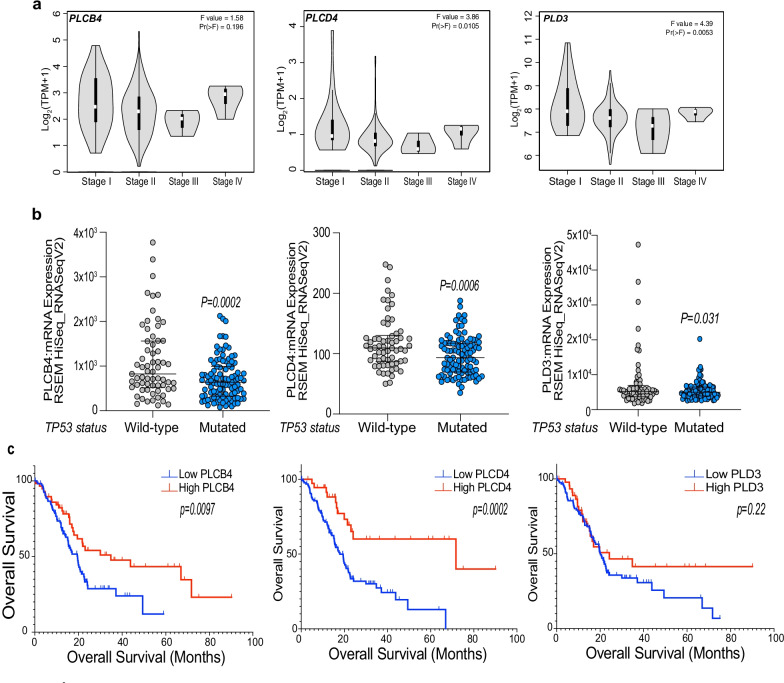
Phospholipases’ expression influences the PDAC-affected patients’ survival. **a** Violin plots showing the RNA levels (Transcript per million, TPM) of PLCB4, PLCD4 and PLD3 across the different stages of pancreatic adenocarcinoma. Source: GEPIA. **b** Scatter dot plots showing the expression of PLCB4, PLCD4 and PLD3 according to the mutation status of p53. The patient cohort was retrieved from TCGA PanCancer Atlas from cBioPortal database. Values are represented as median with interquartile range. *P* values are indicated. **c** Kaplan–Meier survival curve according to the mRNA expression of PLCB4, PLCD4 and PLD3 in PDAC TCGA dataset. *P* values are indicated

By analysing PanCancer genomic data, we then asked whether PLCD4, PLCB4 and PLD3 levels correlate to the pathogenesis of human pancreatic ductal carcinoma. To address this, we performed a bioinformatic analysis of available datasets of human pancreatic adenocarcinoma (PAAC). Interestingly, with the help of Gene Expression Profiling Interactive Analysis (GEPIA), we observed that the expression levels of the three phospholipases underwent a decrease through the different stages of PAAC (Fig. 4a). Next, we sought to determine the relationship of the selected phospholipases with p53 mutational status, which is highly mutated in pancreatic cancer. We therefore analysed the expression levels of PLCD4, PLCB4 and PLD3 in a cohort of 184 patients belonging to the TCGA PanCancer Atlas dataset. Interestingly, PLCD4, PLCB4 and PLD3 mRNA levels correlate with p53 status in PDAC patients (Fig. 4b). These data strongly indicate a biologically relevant p53/PLs axis in the pathogenesis of PDAC. To further extend our study, we next focused on the prognostic significance of this molecular markers. By stratifying the patients’ cohort according to the mRNA expression of PLCD4, PLCB4 and PLD3 (Low, High), we computed a Kaplan–Meier survival analysis. The results indicate that higher expression of the PLs represented a good prognostic factor (Fig. 4c). Thus, the p53/PLs axis displays clinical significance for PDAC pathogenesis and integrated with our lipidomic analysis suggests that p53 mediates a transcriptional programme that influence synthesis and secretion of signalling lysophospholipids. These data can therefore indicate the lysophospholipids as novel mediators of cell-autonomous and non-cell-autonomous tumour suppressive function of p53.

## Discussion

Lipidome reprogramming is observed during cell transformation and tumour progression [35]. Despite a significant effort was invested in understanding the role of lipid metabolism in different types of cancers, its connection with tumour suppressive signalling remains generally elusive. Here, we propose a direct connection between the p53 function and the regulation of non-cell-autonomous lysophospholipids.

We aimed to expand, beyond the control of antioxidant pathways [36–39] and ferroptosis [40, 41], our understanding of the tumour suppressor p53 in the regulation of lipidome. We used a modified version of a mouse PDAC cell line, which recapitulates the progression of human PDAC [18]. By performing mass-spectrometry (MS) based lipidomics, we show that loss of p53 in PDAC cells associates with a global rewiring of their lipidome. Particularly, sphinganine, phosphatidylglycerol and lysophospholipid classes show a significant reduction upon loss of p53. On the contrary, other lipid classes are not comprehensively modulated in cells p53-proficient/deficient. However, an accurate analysis of global unchanged classes reveals that specific lipid species, including diacylglycerols, ceramides and phospholipids, can also be modulated in a p53-dependent manner. These data may reflect the specific adaptation of pancreatic cancer cells to metabolic stress under oncogenic signals (LSL-KRAS^G12D^). Indeed, lysophospholipid scavenging is a particular way of metabolic adaptation documented in several cancer types, both in normoxic and hypoxic conditions. Lysophospholipid scavenging has been shown to be an alternative source of nutrient to sustain the cancer cell proliferation and growth [42, 43].

Lysophospholipids have important biological function as they also act as signalling molecules [44]. Their levels have been associated with cell migration and invasion ability in cancer cells and general regulatory mechanisms by sustaining autocrine and paracrine signals at the basis of tumour-microenvironment (TME) interaction [45–47]. All the aforementioned functions of lysophospholipids are dependent on specific phospholipases, whose activity is under the control of intra- and extra-cellular stimuli [48]. In particular, the family of phospholipases C (PLC) is directly linked to the regulation of the intracellular 1-phosphatidylinositol 4,5-bisphosphate/inositol 1,4,5-trisphosphate (PIP2/IP3) ratio which in turn can strongly modulate PI3K/AKT axis activity, thereby regulating cancer cell gene expression and metabolism [49]. So, we speculate that PLCD4 and PLCB4 might contribute to the PDAC tumorigenesis by influencing PI3K/AKT axis.

Importantly, several classes of lysophospholipids can influence tumour immunity; our data indicate that p53 loss could correlate with a reduction of pro-inflammatory lysophospholipids, suggesting a potential mechanism of immune evasion in cancer. However, metabolic adaptations leading to the lipidome rewiring appear highly cancer type-specific [50, 51]. Hence in our model, the observed decrease of lysophospholipids may be a specific signature of PDAC progression following p53 inactivation. By integrating untargeted lipidomics with the analysis of human cancer database, we have also identified three phospholipases (PLCB4, PLCD4 and PLD3) with a potentially clinical interest in the pathogenesis of PDAC and that might explain the decrease in lysophospholipid species observed in p53-deficient cells.

Tumour cell membranes are highly saturated as compared to normal cells due to the increase of the de novo lipogenesis pathway. Such pathway not only supports the enhanced proliferation rate of cancer cells but also generates membrane lipids which act as scavengers for oxidative stress [52]. Damaged phospholipids are regenerated in the Land’s cycle through a two-step reaction: (1) removal of the damaged acyl chain by the action of phospholipase A (PLA) activity; (2) re-acylation of the generated lysophospholipids by specific acetyltransferases [53, 54]. Although the phospholipases identified in our study do not possess a PLA-like activity, the net increase of lysophospholipid species in p53 proficient cells might support the hypothesis of a regulatory mechanism driven by p53 on Land’s cycle to replenish damaged phospholipids. These observations pave the way to further analyse the activity of pancreatic phospholipases in p53 proficient/deficient PDAC cells.

While future work will be required to dissect the role of specific types and species lysophospholipids in PDAC pathogenesis, our study implicates this class of lipids in p53 tumour suppressive function and suggests their potential role as mediators of remodelling of microenvironment and immunity in p53 inactivated cancers.

## Materials and methods

### Cell culture

Mouse pancreatic inducible KPsh cell line was a kind gift of Prof. S. Lowe and was established has previously described [18]. KPsh cells were grown in DMEM supplemented with 10% fetal bovine serum (FBS) and 2 mM penicillin/streptomycin in presence of 1 ug/ml doxycycline to induce the shRNA targeting *Trp53* mRNA.

### RNA extraction, RT and qPCR analysis

RNA was extracted using RNeasy Mini Kit (Qiagen) according to the manufacturer’s instruction. One microgram of RNA was subsequently reverse transcribed with the SensiFAST cDNA Synthesis kit (Meridian Bioscience, BIO-65054) following the manufacturer’s instructions. qRT-PCR was performed using Fast SYBR Green PCR Master Mix (Applied Biosystems). The relative gene expression was calculated following the 2^−ΔΔct^ method after normalization to mouse TATA-binding protein (TBP). The primers sequences are listed as follows: Plcb4 Fw 5′-GGCCTTTCTGACCAACACAAC-3′, Plcb4 Rev 5′-CTGTTTTCCCTGATGCGAAGG-3′; Plcd4 Fw5′-ATGGACCACCAGGAGCAAAT-3′, Plcd4 Rev 5′- TCTGAAACTCATCCGGCCAT-3′; Pld3 Fw 5′-AAGTAGCAGCCAACGTCTGA-3′, Pld3 Rev 5′-TCCTGGTACATCAGTTTGGGC-3′.

### Lipidomics

For MS-based lipidomics, KPsh cells were cultured in presence or not of doxycycline for 4 days. Cells were harvested and pellets of 1 × 10^6^ cells per replicate were snap-frozen and stored at −80 °C. Five biological replicates per condition were prepared for the analysis via Ultra-high-pressure liquid chromatography coupled to high-resolution tandem mass spectrometry (UHCPL-MS/MS – Vanquish and QExactive, Thermo Fisher, San Jose, CA, USA), as extensively described in prior technical notes [55] or studies on pancreatic cancer [56].

### Bioinformatic analyses

To analyse the expression of PLD3, PLCD4 and PLCB4 across the different stages of Pancreatic Adenocarcinoma, the GEPIA website was used (http://gepia.cancer-pku.cn/about.html [57]).

The cBioportal database was interrogated to retrieve data about human pancreatic adenocarcinoma (http://www.cbioportal.org). Specifically, the TCGA PanCancer Atlas dataset was used for the analysis (cohort: 184 patients).

For Kaplan–Meier survival analysis, the patient cohort from PanCancer dataset was divided into two groups depending on PLD3, PLD4 and PLCB4 expression (Low expression, High expression).

The ChIP-seq analysis was performed by ChIP-Atlas Database (https://chip-atlas.org/peak_browser) and Integrative Genomics Viewer (http://www.broadinstitute.org/igv/) for peaks visualization: ATAC-seq (id: SRX4961722), H3K4me3 (id: SRX3710128), H4ac (id: SRX4384461), H3K9ac (id: SRX8156791). Furthermore, publicly available ChIP-seq data for p53 were reanalysed using the Galaxy tool (https://usegalaxy.org [58]) and are available under the accession number GSE63666.

### Statistical analysis

All graphs and statistical analyses were prepared using GraphPad Prism 8.0 (GraphPad Software Inc.) and MetaboAnalyst 5.0 (https://www.metaboanalyst.ca/home.xhtml). All results are expressed as the mean ± standard deviation (SD). RT-qPCR were analysed by *t*-test (*p < 0.05, **p < 0.01, ***p < 0.001). For Kaplan–Meier analysis, the Mantel-Cox test was applied. All experiments were performed with at least three biological replicates.

## Supplementary Information

**Additional file 1: Figure S1**. Heatmap showing the lipid species significantly affected by p53 loss. For lysophospholipids, only the ten most significantly deregulated species are shown. Cer: Ceramides, DG: Diacylglycerol, LPC, Lysophosphatidylcholine; LPS, Lysophosphatidylserine; PC, Phosphatidylcholine; SPH, Sphinganine; LPE, Lysophosphatidylethanolammine; LPG, Lysophosphatidylglycerol; LPI, Lysophosphatidylinositol; PE, Phosphatidylethanolammine; PG, Phosphatidylglycerol; PI, Phosphatidylinositol; SM, Sphingomyelin; PS, Phosphatidylserine. *p < 0.05; **p < 0.01; ***p < 0.001; ****p < 0.0001. **Figure S2**. **a** Violin plots showing the lipid classes that were not significantly modulated by p53 loss. Lipid abundancies are shown as signal intensities (AU: arbitrary units). N = 5 biological replicates per condition.* P* values are indicated. **b** Heatmap showing global changes of lipids in the conditioned media upon depletion of p53. Lipid abundancies are sown as signal intensities (AU: arbitrary units). N = 5 biological replicates per condition. **Figure S3**. **a** Box plots showing the most significantly affected lysophosphatidylcholine (LPC) species in the conditioned media. Lipid abundancies are shown as signal intensities (AU: arbitrary units). N = 5 biological replicates per condition, *p < 0.05; **p < 0.01; ***p < 0.001. **Figure S4**. **a**–**c** Box plots showing the most significantly affected intracellular lipid species per lysophospholipid class. Lipid abundancies are shown as signal intensities (AU: arbitrary units). N = 5 biological replicates per condition. ****p < 0.0001. LPE, Lysophosphatidylethanolammine; LPS, Lysophosphatidylserine; LPI, Lysophosphatidylinositol; LPG, Lysophosphatidylglycerol.

## Data Availability

Available upon requests.
