# Supplementary figures and images for: p53-driven lipidome influences non-cell-autonomous lysophospholipids in pancreatic cancer
Source: Biol Direct. 2022 Mar 8;17:6. doi: 10.1186/s13062-022-00319-9 (PMC8902766; doi:10.1186/s13062-022-00319-9)

Supplementary Figure 1

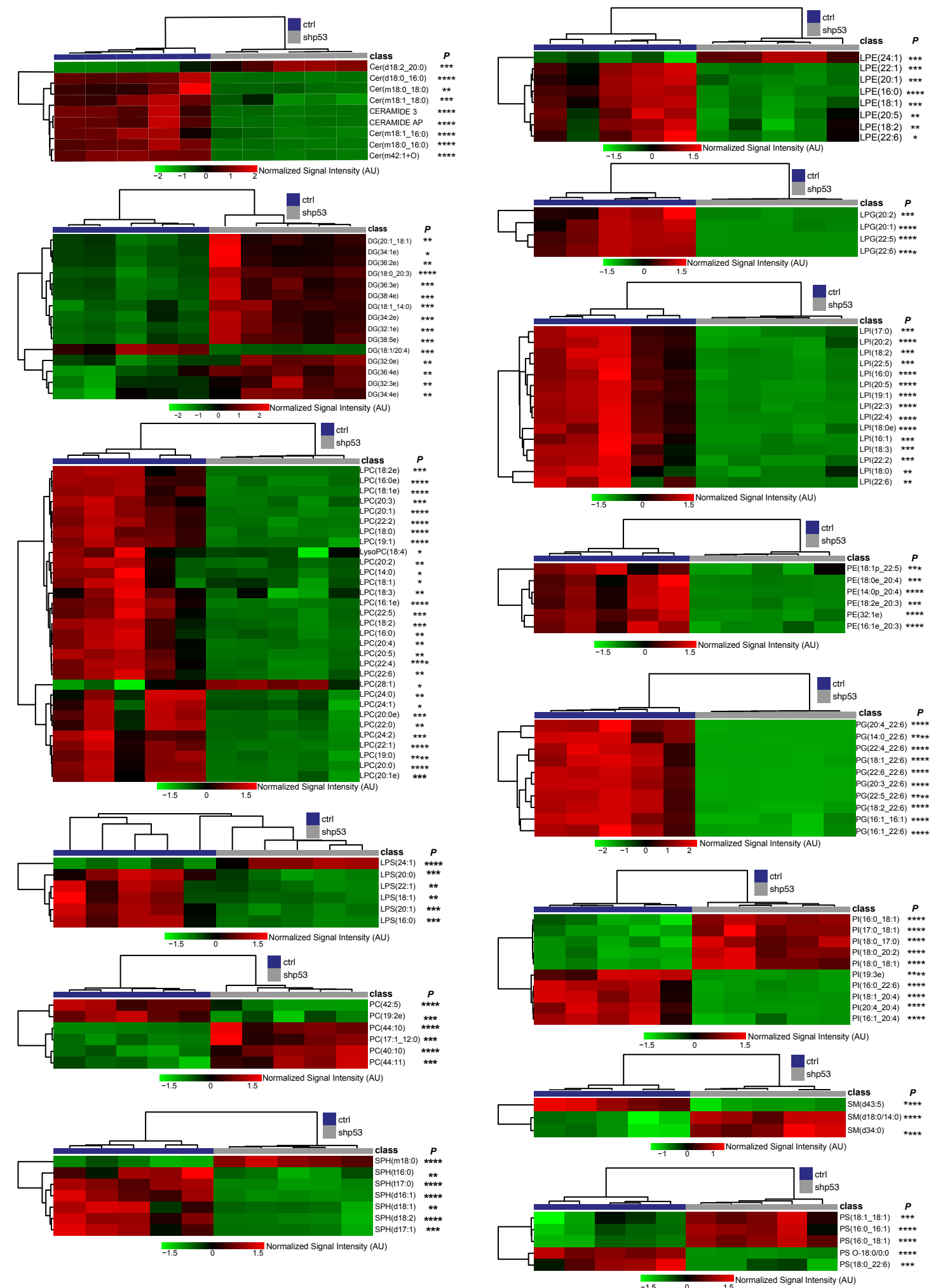

Supplementary Figure 2

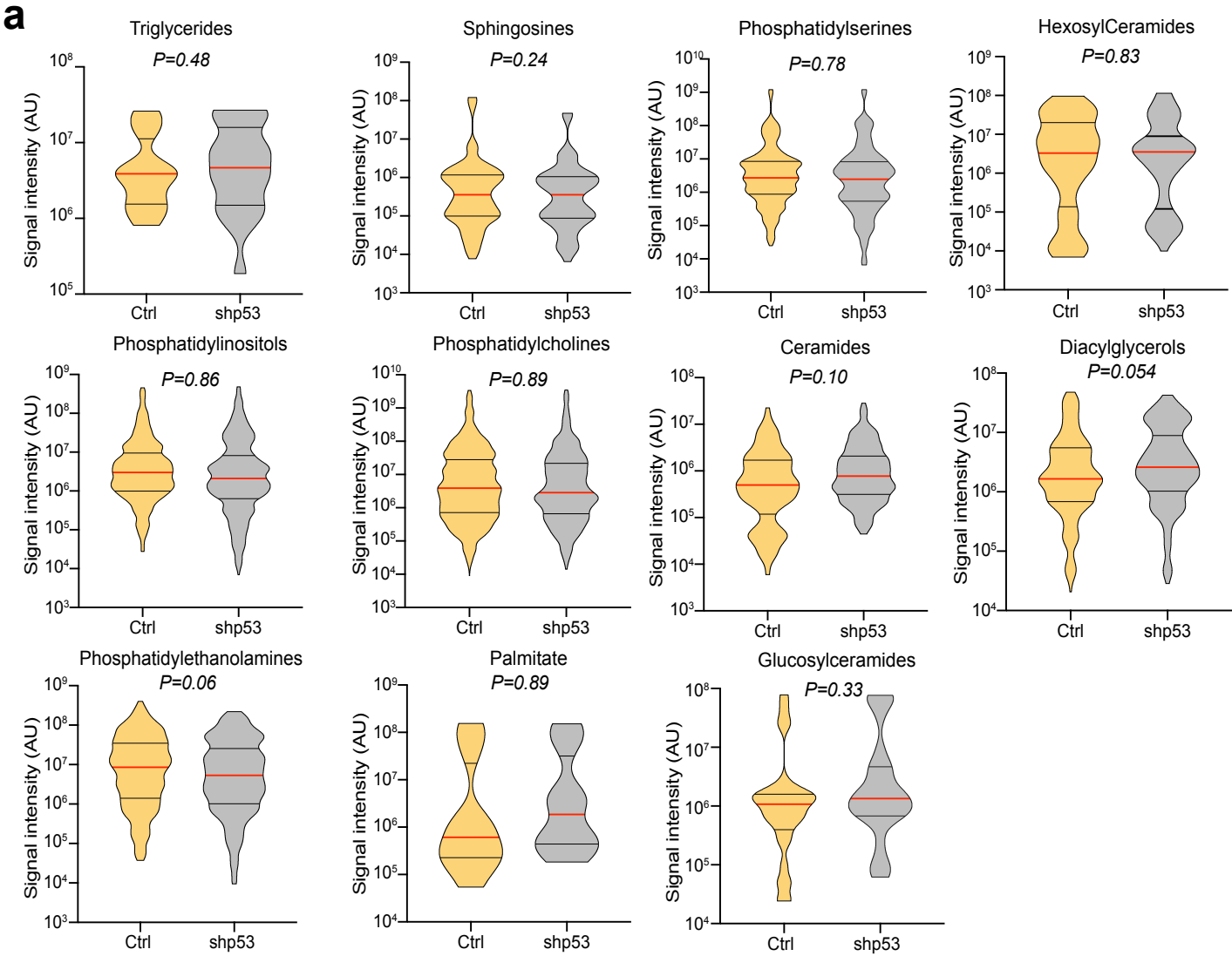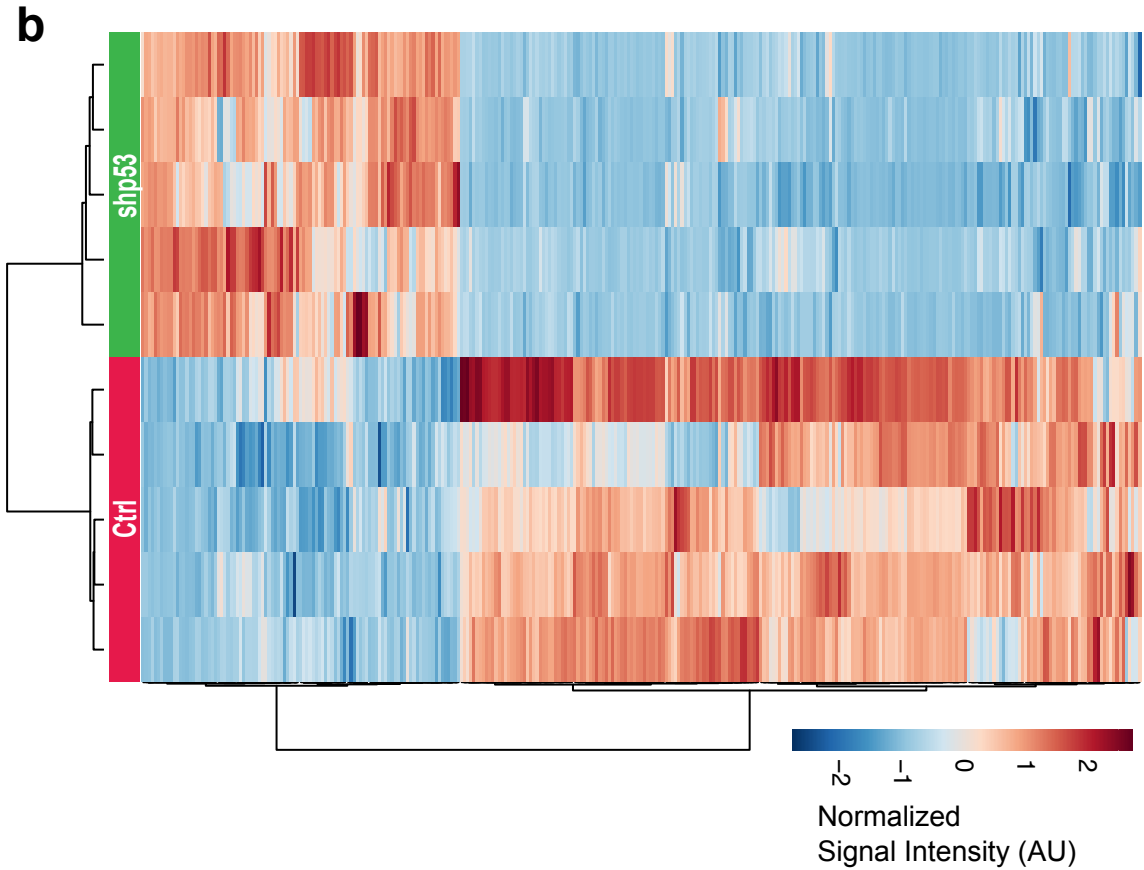

Supplementary Figure 3

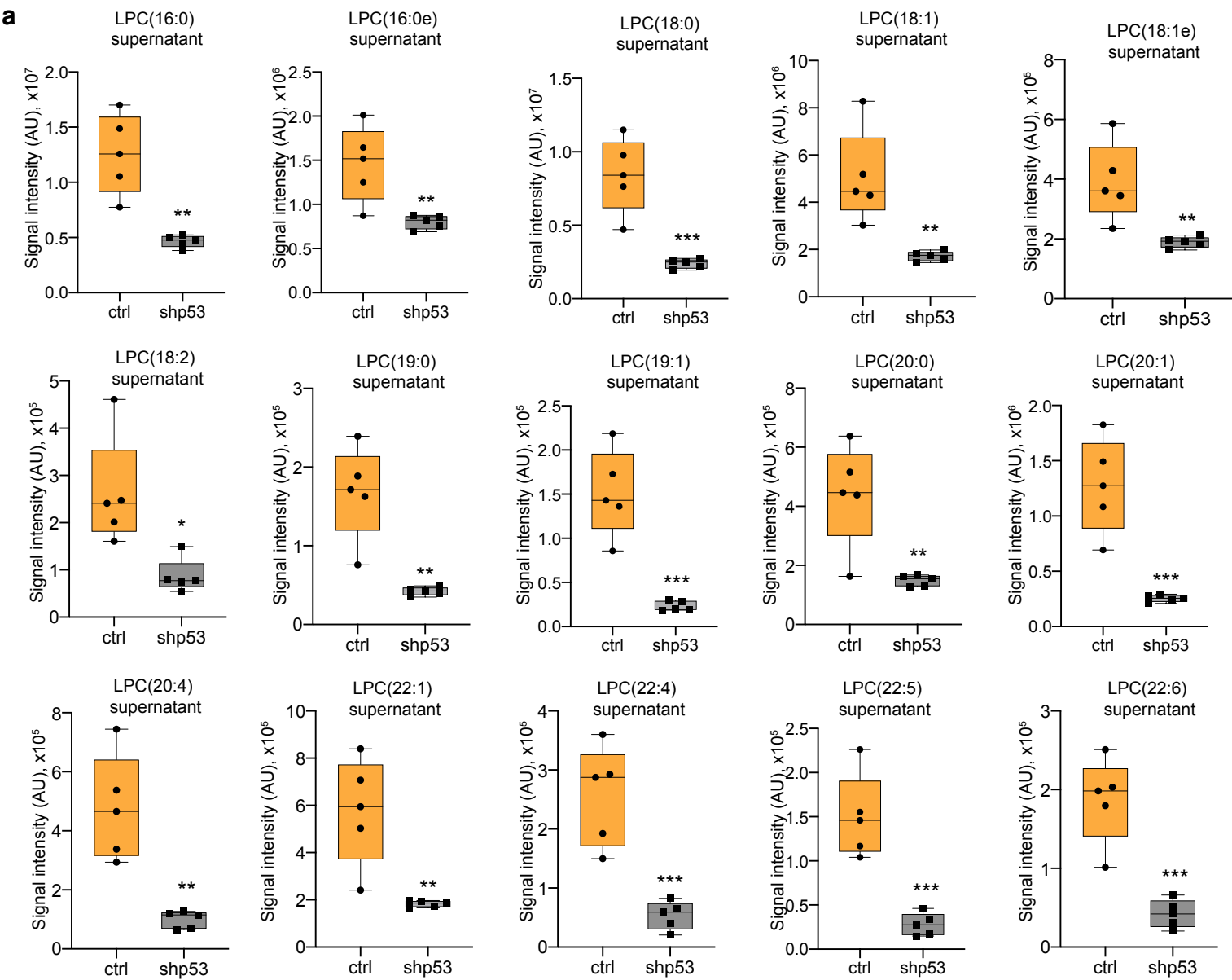

Supplementary Figure 4

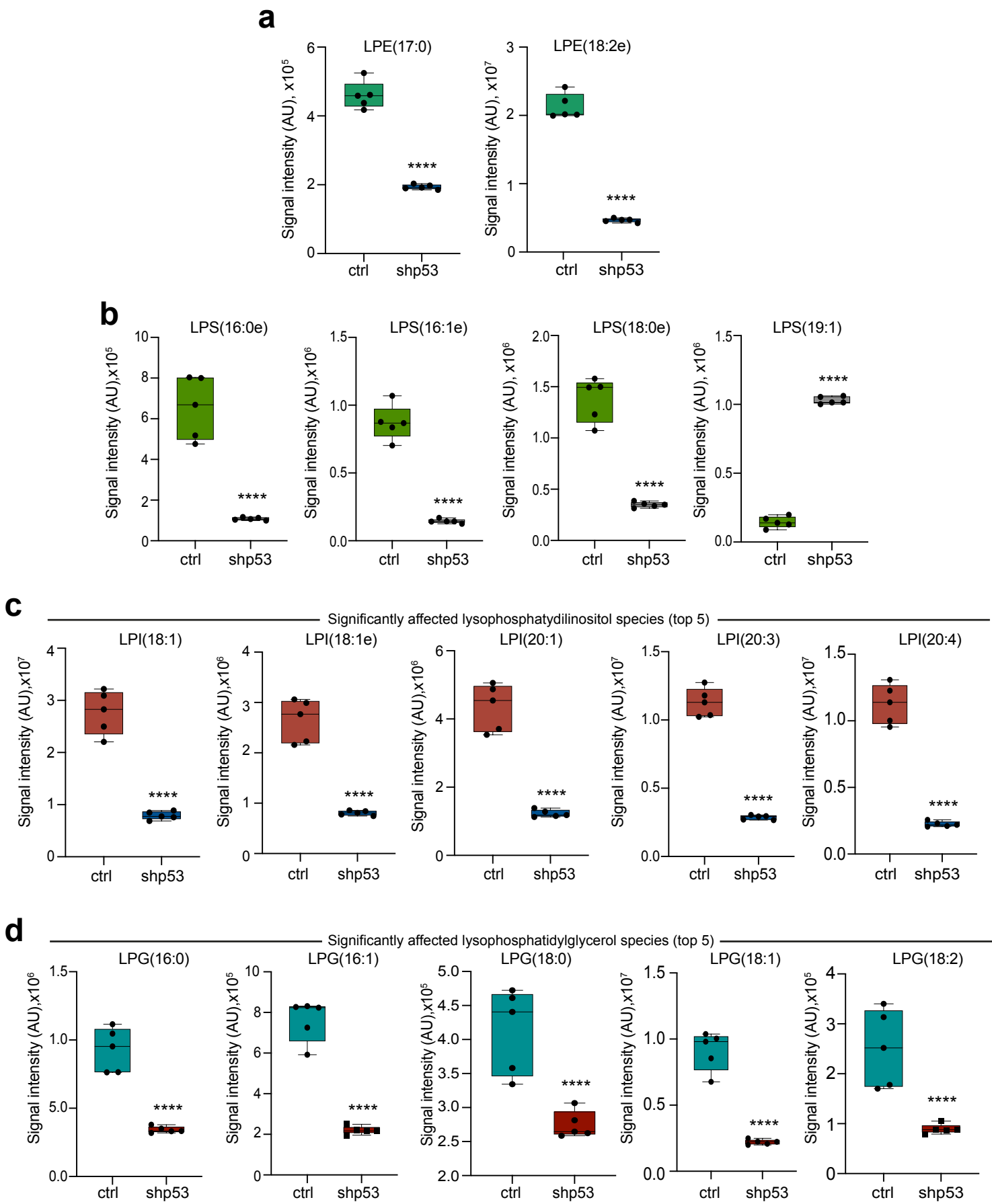

Supplement: Supplementary file 1 — Additional file 1: Figure S1. Heatmap showing the lipid species significantly affected by p53 loss. For lysophospholipids, only the ten most significantly deregulated species are shown. Cer: Ceramides, DG: Diacylglycerol, LPC, Lysophosphatidylcholine; LPS, Lysophosphatidylserine; PC, Phosphatidylcholine; SPH, Sphinganine; LPE, Lysophosphatidylethanolammine; LPG, Lysophosphatidylglycerol; LPI, Lysophosphatidylinositol; PE, Phosphatidylethanolammine; PG, Phosphatidylglycerol; PI, Phosphatidylinositol; SM, Sphingomyelin; PS, Phosphatidylserine. *p < 0.05; **p < 0.01; ***p < 0.001; ****p < 0.0001. Figure S2. a Violin plots showing the lipid classes that were not significantly modulated by p53 loss. Lipid abundancies are shown as signal intensities (AU: arbitrary units). N = 5 biological replicates per condition. P values are indicated. b Heatmap showing global changes of lipids in the conditioned media upon depletion of p53. Lipid abundancies are sown as signal intensities (AU: arbitrary units). N = 5 biological replicates per condition. Figure S3. a Box plots showing the most significantly affected lysophosphatidylcholine (LPC) species in the conditioned media. Lipid abundancies are shown as signal intensities (AU: arbitrary units). N = 5 biological replicates per condition, *p < 0.05; **p < 0.01; ***p < 0.001. Figure S4. a–c Box plots showing the most significantly affected intracellular lipid species per lysophospholipid class. Lipid abundancies are shown as signal intensities (AU: arbitrary units). N = 5 biological replicates per condition. ****p < 0.0001. LPE, Lysophosphatidylethanolammine; LPS, Lysophosphatidylserine; LPI, Lysophosphatidylinositol; LPG, Lysophosphatidylglycerol. [file 13062_2022_319_MOESM1_ESM.pdf]
